# The Relationship Between Pituitary Axis Hormones and All-Cause Mortality in Hospitalized Patients with Chronic Diseases: A Prospective Cohort Study

**DOI:** 10.3390/medicina62030491

**Published:** 2026-03-05

**Authors:** Esin Havare, Güneş Topçu, Pınar Yıldız, Emre Hoca, Hayriye Esra Ataoğlu

**Affiliations:** 1 Department of Internal Medicine, Istanbul Haseki Training and Research Hospital, Istanbul 34265, Turkey; 2 Obstetrics and Gynecology Department, Bahçelievler Medipol Hospital, Istanbul 34196, Turkey; 3 Obstetrics and Gynaecology Department, Şişli Hamidiye Etfal Training and Research Hospital, Istanbul 34371, Turkey

**Keywords:** pituitary axis, mortality, chronic disease, free T3, luteinizing hormone

## Abstract

*Background and Objectives*: The pituitary gland plays a central role in endocrine regulation, and chronic illnesses may disrupt pituitary axis function, potentially influencing clinical outcomes. In this study, we aimed to thoroughly investigate the effects of the pituitary axis on all-cause mortality in patients with chronic diseases. *Materials and Methods*: This prospective observational cohort study included 526 patients with chronic diseases lasting longer than six months who were hospitalized in the Internal Medicine Department of a training and research hospital in Istanbul between 7 May and 30 November 2023. Hormonal parameters were measured within the first 72 h of admission. Demographic characteristics, comorbidities, laboratory findings, intensive care unit (ICU) transfer, and mortality outcomes were recorded. Multivariable binary logistic regression analysis was used to identify independent predictors of mortality. *Results*: Patients who died were significantly older (75.60 ± 12.6 years, *p* < 0.001) and had lower free triiodothyronine (FT3) levels (*p* < 0.001). In hierarchical multivariable logistic regression analysis, increasing age, lower serum albumin levels, immobilization, low FT3 levels, and abnormal luteinizing hormone (LH) levels (defined as values outside sex- and menopausal status-adjusted reference intervals) were independently associated with 6-month mortality. The addition of hormonal parameters significantly improved model performance beyond traditional markers of illness severity. *Conclusions*: Alterations in pituitary axis hormones, particularly deviations in LH levels and reduced FT3 levels, are independently associated with mortality in hospitalized patients with chronic diseases. These findings suggest that pituitary hormone profiles may contribute to the stratification of mortality risk in this population.

## 1. Introduction

The pituitary gland is a fundamental endocrine organ that regulates the secretion of vital hormones in response to hypothalamic signals; it consists of anterior and posterior lobes, with the anterior pituitary producing six major hormones. The body initiates a controlled stress response involving both neural and endocrine mechanisms to maintain energy balance and hemodynamic stability in situations that disrupt homeostasis.

The hypothalamus and pituitary gland are two key players in regulating the stress response. As the primary control center for several neuroendocrine systems, the hypothalamus receives complex sensory inputs from various internal and external stimuli. Combined with endocrine feedback loops, these inputs stimulate the hypothalamus to produce and secrete tropic hormones into the hypophyseal portal system, primarily targeting the anterior pituitary. Highly regulated by these stimulatory or inhibitory hypophysiotropic hormones and various feedback loops, the anterior pituitary produces a series of hormones that act on peripheral glands (such as the thyroid, adrenals, or gonads) or directly target end organs, such as the liver, muscle, and bone [1]. The physiological response of the organism to acute stress and chronic illness is largely shaped by the coordination of the hypothalamic-pituitary axis [1]. Consequently, alterations in pituitary hormone levels are considered significant biological indicators that may determine clinical course and prognosis, particularly in individuals with chronic diseases.

There are five main neuroendocrine axes constituting anterior pituitary function: the somatotropic axis, the thyroid axis, the adrenal axis, the lactotropic axis, and the gonadal axis. Previous studies have demonstrated that these axes are affected during acute and chronic phases of critical illness, and in chronic diseases such as chronic kidney disease [2], chronic obstructive pulmonary disease [3], chronic inflammatory diseases [4], cirrhosis [5], and diabetes mellitus [6], with alterations observed compared with the normal population. Adaptive neuroendocrine alterations occur in acute and chronic disease states. Non-thyroidal illness syndrome, resulting from disruption of the hypothalamic–pituitary–thyroid axis and characterized by reduced FT3 levels, has been associated with increased mortality. However, it remains unclear whether this represents an adaptive response or a marker of poor prognosis [7,8,9,10]. Similarly, the gonadal axis and the hypothalamic–pituitary–adrenal axis are affected in chronic and critical conditions, with disease severity-dependent changes in gonadotropins, cortisol, and prolactin levels. Nevertheless, the impact of these hormonal changes on clinical outcomes and prognosis remains controversial [1].

In the literature, most studies examining the association between hormones and mortality have predominantly evaluated a single hormonal axis [8,9,10,11,12]. Although alterations in thyroid and gonadal axes during acute and chronic illness have been widely described, prior investigations have largely focused on individual hormonal pathways and predominantly ICU populations [11,12]. Prospective evaluations simultaneously assessing multiple pituitary axes in non-ICU hospitalized internal medicine patients with chronic multimorbidity remain limited. Moreover, few studies have applied structured, severity-adjusted multivariable modelling to determine whether early pituitary hormone alterations independently predict 6-month all-cause mortality beyond conventional markers of illness severity. Therefore, the prognostic significance of comprehensive pituitary profiling in ward-based populations is not yet clearly established.

In this study, we aimed to evaluate the association between early pituitary hormone profiles and 6-month all-cause mortality in hospitalized internal medicine patients with chronic diseases. In addition, we examined the relationship between these hormone levels and ICU admission.

## 2. Materials and Methods

### 2.1. Study Design and Patient Population

This prospective observational cohort study utilized data from a tertiary training and research hospital. The study was reviewed and approved by the relevant Institutional Review Board (Ethics Committee Approval No: 56-2023, Date: 29 March 2023). The study was conducted in accordance with the principles of the Declaration of Helsinki, and written informed consent was obtained from all participants.

A total of 526 adult patients (≥18 years) with chronic diseases lasting longer than 6 months who were hospitalized in the Internal Medicine Department were included in the study between 7 May 2023 and 30 November 2023.

Patients younger than 18 years, those receiving treatment for endocrinological disorders, those using steroids, and those receiving medications that suppress the pituitary axis (heparin, rifampin, ketoconazole, phenytoin, benzodiazepines) after hospitalization were excluded. Patients with a history of pituitary surgery, head and neck surgery, radiotherapy, or residual neurological deficits due to head trauma were also excluded. For patients with repeated admissions, only data from the first hospitalization were included in the analysis.

Due to the unavailability of mortality data for three foreign patients, statistical analyses of mortality were performed on 523 patients.

### 2.2. Data Collection

Demographic characteristics, detailed medical history, comorbidities, regular medications, and laboratory results of the included patients were obtained from patient files and the hospital information management system. Patients were grouped by sex as female or male. Since all participants had at least one chronic disease fulfilling the inclusion criteria, an additional multimorbidity variable was constructed to reflect the burden of coexisting chronic conditions beyond the primary admitting diagnosis. Patients were classified as having multimorbidity if they had one or more of the following coexisting chronic conditions: diabetes mellitus, hypertension, heart failure, atrial fibrillation, coronary artery disease, chronic kidney disease, chronic obstructive pulmonary disease, or cerebrovascular disease. Patients without any of these additional conditions were categorized as having no additional comorbidity. These conditions were selected a priori based on their high prevalence in hospitalized internal medicine populations and their well-established associations with increased mortality risk in previous clinical studies. Of the 526 patients included in the study, 483 were classified as having multimorbidity, while 43 had only a single chronic disease without any additional coexisting condition. Patients were also classified according to their mobility status.

Clinical outcomes were documented as either discharge, transfer to the intensive care unit, or all-cause mortality. The length of hospital stay and mortality were analyzed.

### 2.3. Hormonal Assessment

Hormone levels were evaluated from blood samples collected within the first 72 h after hospital admission to reflect early hospitalization status. The hormonal parameters analyzed were thyroid-stimulating hormone (TSH), free triiodothyronine (FT3), free thyroxine (FT4), follicle-stimulating hormone (FSH), luteinizing hormone (LH), cortisol, and prolactin levels. Additionally, total testosterone was recorded in male patients and estradiol in female patients. Reference ranges for all laboratory parameters were based on the values provided by the hospital’s biochemistry laboratory. Categorical classification of LH/FSH levels (normal vs. abnormal) was performed using sex- and age-specific institutional reference intervals that incorporate physiological postmenopausal elevations in women. Serum hormone levels were measured using a fully automated chemiluminescent immunoassay system (Atellica IM 1600 Analyzer, Siemens Healthineers, Erlangen, Germany). All analyses were performed by the direct chemiluminescence method using Siemens Healthineers commercial reagent kits specific to each hormone, in accordance with the manufacturer’s instructions.

### 2.4. Endpoints

The primary endpoint of the study was all-cause mortality occurring within 6 months of hospital admission. The secondary endpoint was transfer to the intensive care unit.

### 2.5. Statistical Analysis

All statistical analyses were performed using IBM SPSS Statistics for Windows, Version 26.0 (IBM Corp., Armonk, NY, USA). Continuous variables were expressed as mean ± standard deviation or median (minimum–maximum), as appropriate, while categorical variables were presented as numbers and percentages. The normality of distribution was assessed using the Kolmogorov–Smirnov test. For comparisons between two groups, Student’s *t*-test was used for normally distributed continuous variables and the Mann–Whitney U test for non-normally distributed variables. Categorical variables were compared using the chi-square test.

Given the number of predictors relative to the number of events, inclusion of all hormonal parameters in the multivariable model could have increased the risk of model overfitting. Therefore, FT3 and LH were selected a priori for multivariable analysis based on their biological relevance in reflecting peripheral thyroid activity and central hypothalamic–pituitary stress response, respectively. Hormonal variables were not selected for multivariable modelling based on univariate statistical significance. Downstream gonadal hormones such as testosterone and estradiol were not included in the final models, as these may be influenced by chronic metabolic status and binding protein dynamics and may not accurately reflect acute endocrine stress-axis dysregulation. Similarly, other hormonal parameters such as FSH, cortisol, and prolactin were not included, as these are either less responsive to acute systemic stress or may exhibit collinearity with inflammatory markers already incorporated into the severity-adjusted models.

Multivariable binary logistic regression analysis was performed to identify independent predictors of 6-month all-cause mortality using a hierarchical (blockwise) modelling strategy. In the first model, age and sex were entered as baseline demographic variables. In the second model, laboratory parameters associated with systemic illness severity (hemoglobin, creatinine, albumin, AST, and CRP) were additionally included. In the third model, immobilization status and multimorbidity status were introduced to account for baseline functional capacity and chronic disease burden. Immobilization status was included as a surrogate marker of frailty and reduced functional reserve, which may independently influence clinical outcomes beyond biochemical indicators of disease severity.

In the final step, FT3 and LH were entered either as continuous variables (Model 4a) or as categorical variables (Model 4b) to evaluate their independent association with mortality. Odds ratios (ORs) with 95% confidence intervals (CIs) were reported. Model performance across sequential blocks was assessed using changes in the omnibus likelihood ratio test (Δχ^2^) and Nagelkerke’s R^2^ values, while model calibration was evaluated with the Hosmer-Lemeshow goodness-of-fit test.

In addition, to examine whether the observed associations were confounded by critical illness severity, a subgroup analysis was performed after excluding patients who required intensive care unit (ICU) admission during hospitalization. The same hierarchical logistic regression strategy was applied in this ward-only cohort. A *p*-value < 0.05 was considered statistically significant.

## 3. Results

### 3.1. Demographic Characteristics

The demographic characteristics of the study population are presented in Table 1. The mean age of patients who died was statistically significantly higher than that of survivors (75.60 ± 12.6 vs. 64.77 ± 16.6 years; *p* < 0.001). There was no significant association between sex and mortality (*p* = 0.421).

### 3.2. Hormone Levels

The relationship between hormone levels and mortality is presented in Table 2. FT3 levels were significantly lower in patients who died (*p* <0.001), while no significant differences were observed between the groups for TSH and FT4 levels. Total testosterone levels were lower in the deceased group (*p* = 0.004), whereas estradiol levels were higher (*p* = 0.033). Cortisol levels were also significantly higher in patients who died (*p* = 0.005). Mean FSH, LH, and prolactin levels were not associated with mortality.

### 3.3. Categorical Analysis of Hormone Levels

**Figure 1:**
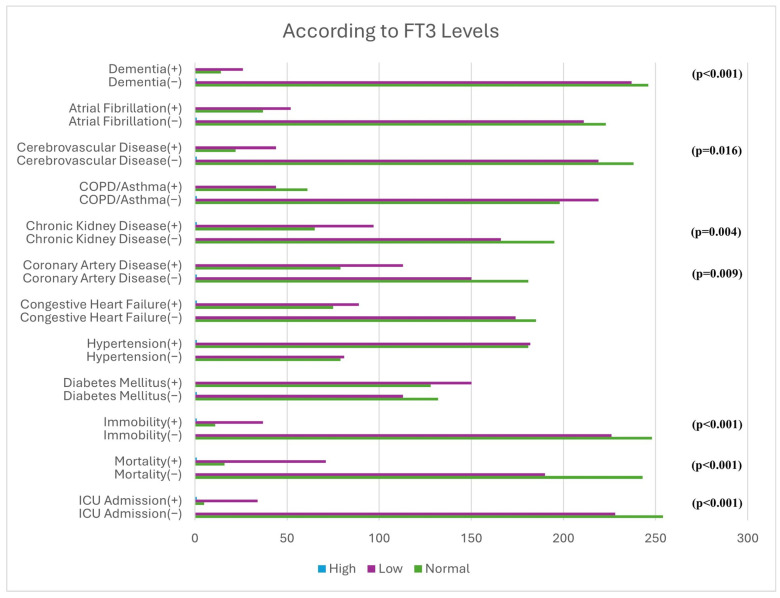
Association of FT3 levels with mortality, ICU admission, and comorbidities.

When hormone levels were categorized, mortality and the frequency of transfer to the intensive care unit were significantly higher in patients with low FT3 levels (Figure 1). The prevalence of immobility, coronary artery disease, chronic kidney disease, cerebrovascular disease, and dementia was also increased in these patients.

**Figure 2:**
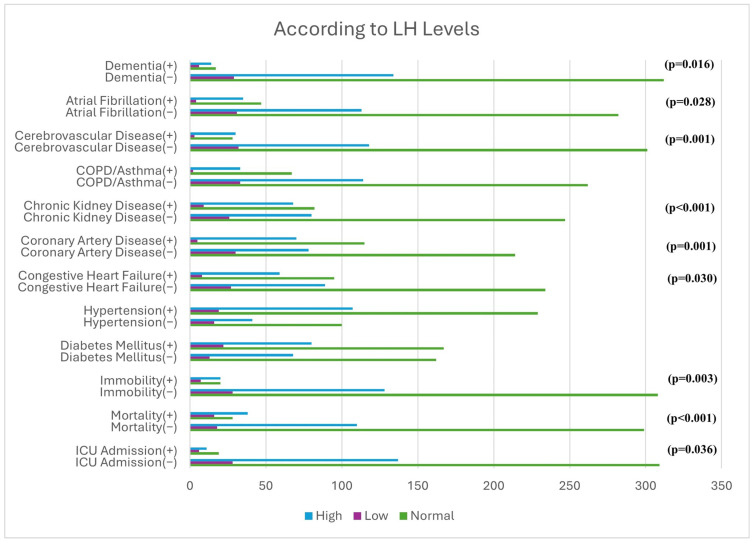
Association of LH levels with mortality, ICU admission, and comorbidities.

Mortality was significantly higher in patients with LH levels outside the normal range (Figure 2). While the frequency of ICU transfer was increased in patients with low LH levels, patients with high LH levels more frequently had congestive heart failure, coronary artery disease, chronic kidney disease, cerebrovascular disease, and atrial fibrillation.

Patients with FSH levels outside the normal range had higher rates of mortality and immobility. Although ICU transfer frequency was higher among patients with elevated prolactin levels, the association between prolactin levels and mortality appeared limited.

**Figure 3:**
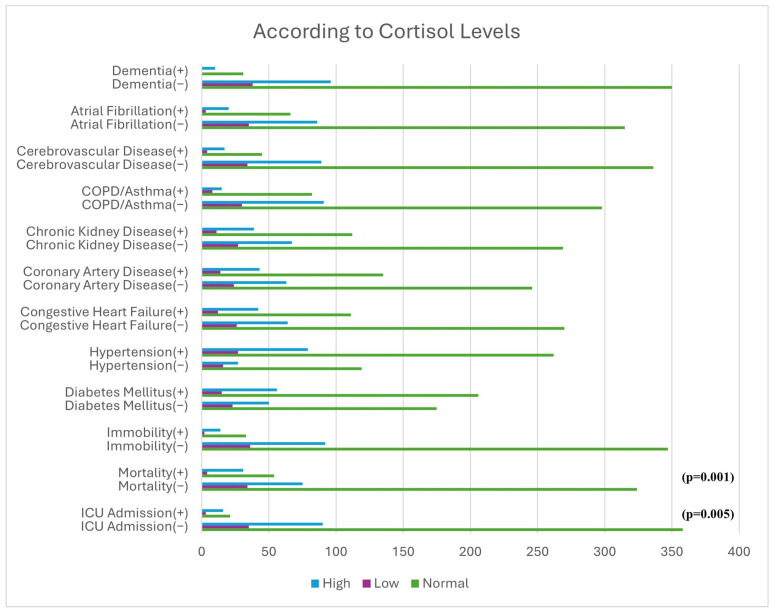
Association of cortisol levels with mortality, ICU admission, and comorbidities.

Mortality and ICU transfer rates were significantly higher in patients with high cortisol levels (Figure 3).

### 3.4. Clinical Characteristics and Mortality

**Figure 4:**
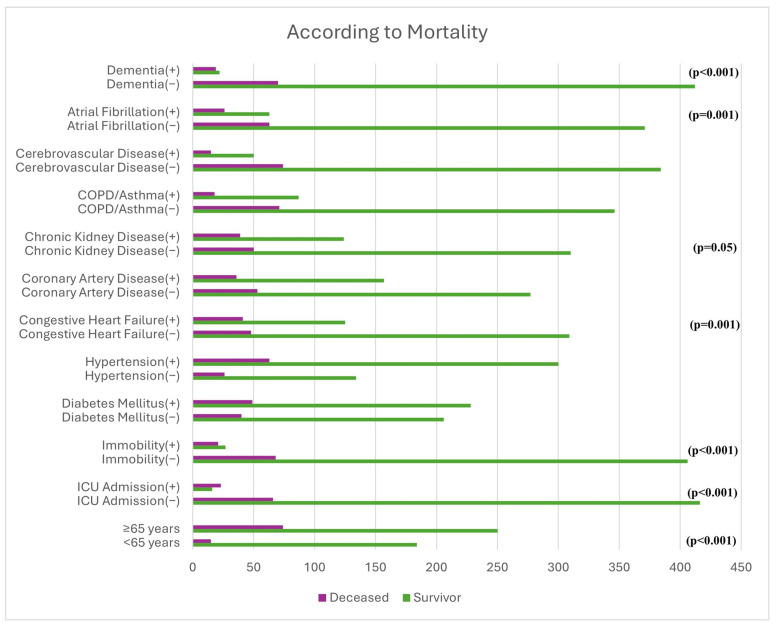
Association of clinical characteristics and comorbidities with mortality.

Mortality was significantly higher among patients aged ≥65 years, those transferred to the ICU, immobilized patients, and those with atrial fibrillation, congestive heart failure, or dementia (all *p* < 0.05). Diabetes mellitus, hypertension, coronary artery disease, COPD/asthma, and cerebrovascular disease were not associated with mortality (Figure 4).

Only parameters showing a statistically significant association with mortality were illustrated graphically to enhance clarity and avoid redundancy.

### 3.5. Multivariate Analysis

In the hierarchical logistic regression analysis performed in the overall cohort (Table 3), increasing age was consistently associated with higher mortality across all models. Lower albumin levels and immobilization status were also independently associated with mortality after adjustment for laboratory markers of systemic illness severity and presence of multimorbidity (Model 3).

When hormonal parameters were introduced as continuous variables (Model 4a), lower FT3 levels remained independently associated with mortality (OR = 0.423, 95% CI: 0.229–0.779; *p* = 0.006), whereas LH levels did not demonstrate a significant association.

In the categorical model (Model 4b), both low FT3 (OR = 2.727, 95% CI: 1.417–5.251; *p* = 0.003) and abnormal LH levels (OR = 3.063, 95% CI: 1.638–5.728; *p* < 0.001) were independently associated with mortality. The addition of categorical hormonal variables resulted in a significant improvement in model performance (Δχ^2^ = 22.010, *p* < 0.001), with an increase in Nagelkerke’s R^2^ from 0.242 to 0.305.

To further evaluate whether these associations were driven by patients with greater illness severity, the analysis was repeated after excluding individuals who required ICU admission during hospitalization (Table 4). In this ward-only cohort, lower FT3 levels remained independently associated with mortality when entered as a continuous variable (OR = 0.376, 95% CI: 0.180–0.785; *p* = 0.009).

Similarly, in the categorical model both low FT3 (OR = 2.531, 95% CI: 1.241–5.161; *p* = 0.011) and abnormal LH levels (OR = 4.421, 95% CI: 2.108–9.273; *p* < 0.001) were independently associated with mortality after adjustment for demographic variables, laboratory markers of illness severity, immobilization status, and multimorbidity burden. The inclusion of categorical hormonal variables significantly improved model performance (Δχ^2^ = 22.835, *p* < 0.001), with Nagelkerke’s R^2^ increasing from 0.194 to 0.274.

These findings suggest that the observed associations between FT3, LH, and mortality are not solely explained by critical illness requiring intensive care support but are also evident among non-ICU hospitalized patients.

## 4. Discussion

In this study, alterations in pituitary axis-related hormone levels were associated with mortality in patients with chronic diseases. In addition to reduced FT3 levels, deviations of LH from physiologically expected ranges—defined using sex- and age-adjusted reference intervals—were independently associated with mortality. Importantly, LH values within the expected range for postmenopausal women were not classified as abnormal, suggesting that this association reflects stress-related dysregulation of the hypothalamic–pituitary–gonadal axis rather than physiological menopausal elevation alone.

Numerous studies in different patient populations have shown that low T3 levels are associated with both short- and long-term mortality [8,9,10,11,12]. In contrast, evidence on T4 and TSH levels and mortality remains inconsistent. While some studies have linked low or high T4 and low TSH with increased mortality, others have failed to demonstrate a significant association between TSH levels and mortality outcomes [11,13], particularly in intensive care patients. Beyond its role as a biomarker of systemic illness, triiodothyronine (T3) exerts direct effects on cellular energy metabolism, mitochondrial function, and immune regulation. T3 plays a key role in maintaining mitochondrial oxidative phosphorylation and ATP production at the cellular level [14]. Reduced T3 levels have been associated with impaired mitochondrial activity, decreased cardiac contractility, and vascular dysfunction, all of which may contribute to organ dysfunction in acutely ill patients [15]. Furthermore, T3 has been shown to modulate innate and adaptive immune responses through its effects on macrophage activation, cytokine signaling, and T-cell differentiation [16]. In line with the existing literature, our findings confirm a significant association between low FT3 levels and mortality, whereas no independent effect of T4 or TSH levels on mortality was observed. These findings suggest that T3 may represent a more sensitive prognostic biomarker. Importantly, in our subgroup analyses restricted to patients who did not require ICU admission, FT3 remained independently associated with mortality after adjustment for markers of illness severity. This suggests that reduced FT3 levels may not merely reflect advanced systemic illness but could represent a broader dysregulation of metabolic homeostasis in hospitalized patients with chronic diseases.

Similar to previous reports, patients with low T3 levels in our cohort more frequently exhibited comorbid conditions such as coronary artery disease, chronic kidney disease, dementia, and cerebrovascular disease, and higher rates of immobility and ICU transfer [17,18,19,20,21,22,23].

Disruption of pituitary function is often accompanied by marked alterations in gonadal hormone levels. Both hypogonadotropic and hypergonadotropic patterns have been described during hospitalization in patients with chronic diseases [1,24]. The prominence of these changes, particularly in the elderly and during acute illness, suggests a potential prognostic role for the gonadal axis [25,26,27,28].

In a study of hospitalized COVID-19 patients with respiratory failure, low LH and testosterone levels in men and low LH and FSH levels in women were associated with increased mortality [29].

The literature indicates that both elevated and reduced estrogen levels may be associated with mortality in men and women, and that gonadotropin deficiency represents an important risk factor for increased mortality, independent of other hormonal deficiencies [30,31,32,33].

In our study, abnormal LH levels were independently associated with mortality. In addition, LH levels are known to exhibit dynamic changes during acute illness, with initial elevations followed by suppression in prolonged stress states. As hormonal measurements were performed within the first 72 h of admission, the observed variability in LH levels may reflect heterogeneity in the duration or phase of illness before hospitalization. Because both low and high LH values were grouped under the “abnormal” category, this classification may capture heterogeneous stress-related pituitary–gonadal responses rather than a single unified pathophysiological mechanism. Therefore, the association between abnormal LH levels and mortality should be interpreted cautiously and may be more indicative of a systemic stress response rather than a direct prognostic mediator.

Although categorical modelling of LH improved overall model fit, this does not necessarily indicate statistical superiority over continuous modelling. LH was not independently associated with mortality when analysed as a continuous variable, suggesting that the observed association may reflect non-linear or threshold effects rather than a simple linear relationship.

In univariate analyses, low testosterone, low and high FSH levels, and low estradiol levels were associated with mortality; however, these downstream gonadal hormones were not included in the final multivariable models due to their susceptibility to chronic metabolic status, sex hormone-binding protein dynamics, and delayed response to acute systemic stress. Therefore, these parameters may not accurately reflect acute hypothalamic–pituitary–peripheral axis dysregulation during severe illness.

Consistent with the literature, patients with elevated LH levels showed a higher prevalence of congestive heart failure [34], coronary artery disease [35], chronic kidney disease [36], cerebrovascular disease [35,37], and atrial fibrillation [38,39]. An increased frequency of dementia was also observed in patients with abnormal LH levels.

The literature indicates that both high and low cortisol levels are associated with mortality [40,41,42]. Evidence regarding the relationship between prolactin levels and cardiovascular or all-cause mortality remains conflicting. While some studies have reported increased risk with elevated prolactin levels [43,44], others have found no significant association, and certain populations have even demonstrated increased cardiovascular risk with low prolactin levels [45,46]. In our study, although higher cortisol and prolactin levels were associated with ICU requirement and mortality in univariate analyses, these hormonal responses may represent non-specific activation of the stress response and exhibit substantial overlap with inflammatory pathways already accounted for in the severity-adjusted models. For this reason, inclusion of these variables in the final multivariable models was avoided in order to reduce model overfitting and multicollinearity. In light of these findings, as prolactin is influenced by stress, medication use, and comorbidities, the observed association between prolactin and mortality may represent a secondary phenomenon rather than a direct etiological factor.

Overall, these findings should be interpreted within the context of the existing literature describing neuroendocrine adaptations during systemic illness. Alterations of the thyroid and gonadal axes during critical illness have been well documented, particularly in intensive care and septic populations. In contrast, our study evaluated a non-ICU internal medicine cohort composed predominantly of patients with chronic multimorbidity, with hormone measurements obtained within the first 72 h of admission and all-cause mortality assessed within 6 months. By simultaneously examining multiple pituitary axes and demonstrating independent associations with mortality beyond conventional severity markers, our findings add further clinical context to previously reported ICU-based observations.

### Strengths and Limitations

The strengths of the study are its prospective observational design and a relatively large sample size of 526 patients, encompassing a large and heterogeneous population. These features enabled a robust and statistically reliable assessment of the associations between hormonal parameters and mortality. Compared with the limited number of studies examining the prognostic role of pituitary hormones in non-ICU patients with chronic diseases, this study, focusing on patients managed in an Internal Medicine ward, contributes to the literature with its unique perspective and offers insights potentially relevant for clinical practice.

While endocrine stress responses have been well characterized in critical illness, our study extends these observations to a non-ICU internal medicine population with chronic multimorbidity. By prospectively evaluating multiple pituitary axes simultaneously and applying a structured, severity-adjusted multivariable modelling strategy, we demonstrate that early alterations in selected pituitary hormones are independently associated with 6-month mortality beyond conventional markers of illness severity. In this context, our findings provide clinically relevant evidence supporting the potential role of comprehensive pituitary hormone profiling in mortality risk stratification outside the ICU setting.

The main limitations of this study include the lack of detailed information regarding menstrual cycle phases in menstruating women and the inability to standardize timing for FSH and LH measurements, as blood samples were obtained within the first 72 h of hospital admission. However, premenopausal women constituted only a small proportion of the study population (7.6%), which reduces the likelihood that this limitation had a substantial impact on the overall results. In addition, hormone measurements obtained during the acute phase of illness may not fully reflect chronic hormonal status.

Furthermore, given the observational nature of the study, residual confounding due to unmeasured illness severity factors may persist despite multivariable adjustment. The reported associations should be interpreted cautiously and not as evidence of causality.

## 5. Conclusions

In conclusion, alterations in pituitary axis-related hormone levels were independently associated with mortality among patients with chronic diseases. Deviations from sex- and age-adjusted physiological LH reference ranges, together with reduced free triiodothyronine (FT3) levels, remained significantly associated with increased all-cause mortality. These findings highlight the potential value of LH and FT3 as markers for mortality risk stratification. Future studies may explore whether composite risk scores incorporating pituitary axis hormone levels could improve early mortality prediction and clinical decision-making in hospitalized patients with chronic diseases.

## Figures and Tables

**Table 1:** Association between demographic characteristics and mortality.

Variable	Survivors	Non-Survivors	*p* Value
Age (years)	64.77 ± 16.6	75.60 ± 12.6	<0.001
Sex (F/M)	230/204	43/46	0.421

**Table 2:** Association between hormone levels and mortality.

Parameter	Survivors	Non-Survivors	*p* Value
TSH (mU/L)	1.83 ± 1.6	1.89 ± 1.8	0.742
FT3 (ng/L)	2.11 ± 0.5	1.66 ± 0.6	<0.001
FT4 (ng/L)	12.51 ± 2.6	12.41 ± 2.8	0.737
Total testosterone (µg/L)	3.15 ± 2.1	2.07 ± 2.4	0.004
Prolactin (µg/L)	17.57 ± 19.6	19.29 ± 14.1	0.441
Cortisol (µg/dL)	13.64 ± 5.9	17.18 ± 11.4	0.005
Estradiol (ng/L)	28.27 ± 33.7	42.48 ± 65	0.033
FSH (U/L)	25.08 ± 25.7	25.17 ± 24.4	0.977
LH (U/L)	20.41 ± 18.5	20.58 ± 20	0.937

TSH: thyroid-stimulating hormone; FSH: follicle-stimulating hormone; LH: luteinizing hormone.

**Table 3:** Hierarchical logistic regression models for 6-month mortality.

Variable	Model 1 OR (95% CI)	*p*	Model 2 OR (95% CI)	*p*	Model 3 OR (95% CI)	*p*	Model 4a (FT3 & LH Continuous) OR (95% CI)	*p*	Model 4b (FT3 & LH Categorical) OR (95% CI)	*p*
Age (per year)	1.057 (1.035–1.080)	<0.001	1.046 (1.025–1.068)	<0.001	1.042 (1.021–1.063)	<0.001	1.039 (1.018–1.060)	<0.001	1.030 (1.009–1.052)	0.005
Gender (male)	1.525 (0.925–2.515)	0.098	1.408 (0.831–2.383)	0.203	1.428 (0.837–2.435)	0.191	1.533 (0.864–2.721)	0.144	0.957 (0.517–1.771)	0.890
Hemoglobin	–	–	0.895 (0.789–1.015)	0.083	0.890 (0.784–1.011)	0.073	0.886 (0.779–1.009)	0.069	0.888 (0.777–1.015)	0.082
Creatinine	–	–	1.095 (0.921–1.301)	0.304	1.125 (0.948–1.336)	0.178	1.087 (0.907–1.301)	0.366	1.013 (0.835–1.229)	0.894
Albumin	–	–	0.891 (0.844–0.940)	<0.001	0.905 (0.857–0.956)	<0.001	0.933 (0.880–0.990)	0.022	0.938 (0.885–0.994)	0.030
AST	–	–	1.000 (0.999–1.001)	0.967	1.000 (0.999–1.001)	0.920	1.000 (0.999–1.001)	0.745	1.000 (0.999–1.001)	0.669
CRP	–	–	1.001 (0.997–1.006)	0.476	1.001 (0.997–1.006)	0.517	1.000 (0.996–1.005)	0.969	1.001 (0.997–1.005)	0.666
Immobilization	–	–	–	–	2.846 (1.356–5.971)	0.006	2.817 (1.333–5.956)	0.007	2.300 (1.081–4.896)	0.031
Multimorbidity (≥1 additional chronic condition)	–	–	–	–	0.261 (0.090–0.756)	0.013	0.348 (0.115–1.049)	0.061	0.293 (0.098–0.876)	0.028
FT3 (continuous)	–	–	–	–	–	–	0.423 (0.229–0.779)	0.006	–	–
LH (continuous)	–	–	–	–	–	–	0.999 (0.983–1.014)	0.863	–	–
Low FT3 (categorical)	–	–	–	–	–	–	–	–	2.727 (1.417–5.251)	0.003
Abnormal LH (categorical)	–	–	–	–	–	–	–	–	3.063 (1.638–5.728)	<0.001
Nagelkerke R^2^	0.120		0.221		0.242		0.268		0.305	
Δχ^2^ (vs. previous)	–		32.925	<0.001	7.372	0.007	8.932	0.011	22.010	<0.001

Model 1: Adjusted for age and gender; Model 2: Additionally adjusted for hemoglobin, creatinine, albumin, AST, and CRP; Model 3: Additionally adjusted for immobilization status and multimorbidity burden; Model 4a: Additionally adjusted for continuous FT3 and LH; Model 4b: Additionally adjusted for categorical FT3 and LH.

**Table 4:** Hierarchical logistic regression models for 6-month mortality in patients who did not require ICU admission during hospitalization.

Variable	Model 1 OR (95% CI)	*p*	Model 2 OR (95% CI)	*p*	Model 3 OR (95% CI)	*p*	Model 4a (FT3 & LH Continuous) OR (95% CI)	*p*	Model 4b (FT3 & LH Categorical) OR (95% CI)	*p*
Age (per year)	1.058 (1.033–1.083)	<0.001	1.048 (1.023–1.073)	<0.001	1.049 (1.024–1.074)	<0.001	1.043 (1.019–1.069)	0.001	1.034 (1.009–1.060)	0.007
Gender (male)	1.893 (1.072–3.343)	0.028	1.823 (1.013–3.278)	0.045	1.841 (1.017–3.333)	0.044	2.293 (1.188–4.426)	0.013	0.985 (0.485–2.000)	0.967
Hemoglobin	–	–	0.899 (0.783–1.032)	0.130	0.900 (0.782–1.034)	0.137	0.896 (0.777–1.033)	0.131	0.908 (0.782–1.054)	0.205
Creatinine	–	–	1.139 (0.945–1.373)	0.172	1.184 (0.979–1.430)	0.081	1.120 (0.916–1.369)	0.269	1.055 (0.853–1.305)	0.620
Albumin	–	–	0.920 (0.865–0.977)	0.007	0.931 (0.876–0.990)	0.023	0.967 (0.904–1.034)	0.325	0.971 (0.909–1.037)	0.386
AST	–	–	1.000 (0.997–1.002)	0.736	1.000 (0.998–1.002)	0.754	1.000 (0.997–1.003)	0.763	1.000 (0.998–1.001)	0.607
CRP	–	–	1.000 (0.995–1.005)	0.964	1.000 (0.995–1.005)	0.896	0.999 (0.994–1.005)	0.813	1.000 (0.995–1.005)	0.916
Immobilization	–	–	–	–	1.993 (0.827–4.805)	0.125	1.762 (0.726–4.276)	0.211	1.425 (0.570–3.562)	0.448
Multimorbidity (≥1 additional chronic condition)	–	–	–	–	0.409 (0.118–1.416)	0.158	0.427 (0.119–1.530)	0.191	0.534 (0.145–1.970)	0.347
FT3 (continuous)	–	–	–	–	–	–	0.376 (0.180–0.785)	0.009	–	–
LH (continuous)	–	–	–	–	–	–	1.007 (0.990–1.024)	0.413	–	–
Low FT3 (categorical)	–	–	–	–	–	–	–	–	2.531 (1.241–5.161)	0.011
Abnormal LH (categorical)	–	–	–	–	–	–	–	–	4.421 (2.108–9.273)	<0.001
Nagelkerke R^2^	0.118		0.177		0.194		0.223		0.274	
Δχ^2^ (vs. previous)	–		16.303	0.006	4.618	0.099	8.244	0.016	22.835	<0.001

## Data Availability

The data presented in this study are available on request from the corresponding author due to privacy/ethical restrictions.
